# Reawakening of Indigenous matriarchal systems: A feminist approach to organizational leadership

**DOI:** 10.1177/08404704231210255

**Published:** 2023-11-11

**Authors:** Courtney Defriend, Celeta M. Cook

**Affiliations:** 1 1763 Royal Roads University, Victoria, British Columbia, Canada.; 2 8205 University of Victoria, Victoria, British Columbia, Canada.

## Abstract

Leadership models that uphold feminist qualities of mutuality, collaboration, and distribution of power can foster organizational and community success. Utilizing a systems perspective grounded in land-based analogies can assist with understanding the diversity and strength that come from entire ecosystems around wicked social issues. While Indigenous leadership models have supported such perspectives since time immemorial, current and ongoing acts of colonialism driven by patriarchal systems and violent gender-based policies and procedures have eroded matriarchal leadership models that sustained what is now known as Canada for generations. Reflections of two evolving Indigenous women in leadership note the opportunities to reawaken matriarchal values in organizational and community leadership as a powerful act of reconciliation.

## Introduction

Matriarchal systems of leadership are common in the origins of First Nations communities across the Pacific Northwest Coast. These true matriarchal systems not only welcome women in leadership roles, they are rooted in the deeper concept that women are direct reflections of the climate, land, and waters. It is no coincidence that the earth is commonly referred to as the “mother” based on her infinite abilities to provide life and longevity, which is a reciprocal process between humans, creatures, and the environment.^1^ Matriarchs represent how interdependent ecosystems form the wholistic aspects of personal, community, and universal wellness. Women were once seen as the conduit for healthy and strong systems. However, their role in systems of health and well-being has been eroded by colonial practices and policies grounded in settler patriarchy.^2^ As organizations begin to recognize the impacts of colonization on their leadership structures and the broader health system, a powerful act of reconciliation and decolonization is supporting the reawakening of feminine structures in organizations.

## Settler colonialism and gender-based violence

Indigenous-specific racism is an output of settler colonialism and often goes unchallenged in the dominant cultural norms of leadership practice.^3^ Policies and practices such as the *Indian Act*, Indian residential schools, Indian hospitals, and the child welfare system were created to eradicate and assimilate Indigenous Peoples and their ways of knowing and being.^3,4^ The settler systems and structures enforced sexist, racist ideals that shaped the interlocking systems of oppression that continue to influence the health and well-being of Indigenous Peoples today.^5^ Women and 2SLGBTQ+ relatives are overrepresented in social emergencies, including the toxic drug crisis.^2^

Patriarchal perspectives on societal structures led to the suppression of matrilineal kinship. Many First Nations used matrilineal systems for clan membership, with men marrying in to a woman’s family.^6^ The Canadian government implemented fraternal lineage within the *Indian Act,* and created legislation to determine who could be an Indian: women lost their status and rights if they were widowed, left by their husband, or if they married a non-Indian man, regardless of her ancestors. Settler colonial perspectives included heteronormative gender roles, viewing women as subservient to men. Only men could hold positions of power, and women were deemed incapable of participating in political decision-making. Moreover, while Indigenous research and oral histories suggest many communities had important social roles for people of diverse gender identities, the categories of gender, sex, and sexuality most commonly used in dominant society were introduced through colonial processes failing to recognize trans, non-binary, and two spirit people.^6^

The disruption of cultural and traditional roles within communities has created conditions for gender-based violence. Gender-based violence is inherent in settler colonial logic and was a prominent weapon in the attempted elimination of Indigenous Peoples. Residential and day schools perpetuated negative gender stereotypes and imposed homophobic and transphobic beliefs.^7^ Most children within the residential school system were neglected and exposed to abuse, with many experiencing acts of physical, emotional, or sexual violence.^8^ The general state of Indigenous women’s health in Canada including fertility, mental wellness, and general susceptibility to complex and chronic illness have linkages to the violence that colonial systems have projected onto Indigenous Peoples and traditional lands.^4^ Moreover, Indigenous women and 2SLGBTQ+ people face greater instances of physical violence, as recognized in the Missing and Murdered Indigenous Women and Girls movement.^2^

Healthy people create healthy systems. Working to improve the health of Indigenous women and 2SLGBTQ+ people requires reflection on how structures and systems were established, and how they continue to inflict Indigenous-specific racism and gender-based violence. Resurgence of tradition and femininity in our leadership approaches can influence positive change. Correcting power in hierarchy can help to create safer, more welcoming spaces for Indigenous women and 2SLGBTQ+ people to step into leadership roles.

## Unpacking structural violence in leadership

Since the time of contact, corporate and colonial laws, systems, and protocols championed values that starkly contradict Indigenous ways of knowing and being. Suppression of Indigenous knowledge and cultural practices allowed harmful processes to overtake land and damage communities.^7,9^ Capitalist, extractive models prioritized wealth and profit over community well-being. In addition to economic and corporate models that continue to exploit what is referred to as “our mother,” Indigenous Peoples were targeted primarily through their children to indoctrinate patriarchal models of hierarchy and power that settled in what is now known as Canada.^7^ The loss of children holds additional emphasis for women as life-givers, whose purpose is to foster the generations to come.

The devaluing of Indigenous ways of knowing and being were instrumental to the development of Canada, wherein the oppression to Indigenous Peoples continues to play out in Indigenous communities.^2,10^ For example, the violence and oppression endured through residential schools, the potlach ban, and the welfare system are often channelled against each other, wherein community, relatives, and family systems continue to treat each other with competition or disrespect.^7,10,11^ This is commonly referred to as lateral violence. Lateral violence can keep people in cycles of violence, as unprocessed trauma prevents healthy relationships in workplaces and communities.^8^ Feelings of inferiority can lead people to take on learnt qualities of their oppressor and find ways to acquire and abuse power over others.

Notably, the elected chief and council systems developed by the Canadian Government remain a common structure in First Nations communities in Canada today. At one time, the electoral systems were limited to male-only rosters.^8^ Such systems often negate the hereditary or matriarchal systems developed to lead communities in such a way that is reciprocal with the health of the land and the seven generations that came before as well as the seven generations to follow those in leadership positions.^2,12^ In some instances, maintaining a hierarchal system allows for misuse of power and exertion of control. Continuing to draw on hierarchal systems that exploit people through top-down assertions of power prevents acts of resilience to wicked problems that require responsiveness and innovation.^12^

## Learning from our surroundings

Indigenous models of leadership that uphold inclusivity, embodiment of culture, and social inclusion have gained traction in recent approaches to mainstream leadership based on their capacity to create meaningful transformation and organizational sustainability.^13^ Some Indigenous leadership models have been described to simultaneously promote individualistic and collective paradigms. Sustainability, identity, and uniqueness can be fostered in a diverse and collaborative environment. Similar to our ecosystems, Indigenous leadership upholds the cyclical, organic, and process-based considerations that are reciprocal with each organism present within the system. This perspective is relational and grounded in culture and protocol.^14^ Such Indigenous leadership systems were upheld and asserted through their matriarchal structures.

As author CD reflects: *One of the most valuable teachings I apply in my leadership role is the power of sitting in circle. I learnt this from my mother. The circle is a strong figure, having no beginning, no end, and is completely balanced. In fact, an imbalanced location of power can compromise its overall strength. Everyone sits at the same level and like nature, it will always move through to completion in its own time. In my own leadership, I try to apply wholeness; taking the time we need to naturally run our course; patience, process, cycles, and community.*

The biologic and cellular make up of creatures that belong to certain territories contribute to instinctual behaviours such as migration and food sources.^15^ Human beings have similar gifts within their biology. While such concepts were known and applied to leadership structures in Indigenous communities, western science is finally catching up. The uncovering of the fascinating systems that trees participate in as some of the largest organisms on the planet highlights the sophistication that mother earth offers as emblems of healthy networks and intergenerational success. In most circumstances, a tree that stands on its own without reciprocating with its neighbours, soil, and general ecosystem will be much more vulnerable to the unpredictable weather it will endure in its lifetime.^16^ Considering the strength in diversity, every organism in a successful ecosystem works together. While some may seem to play larger roles than others do, even the tiniest contributors actualize the sustainability of the forest.

**Figure 1.**
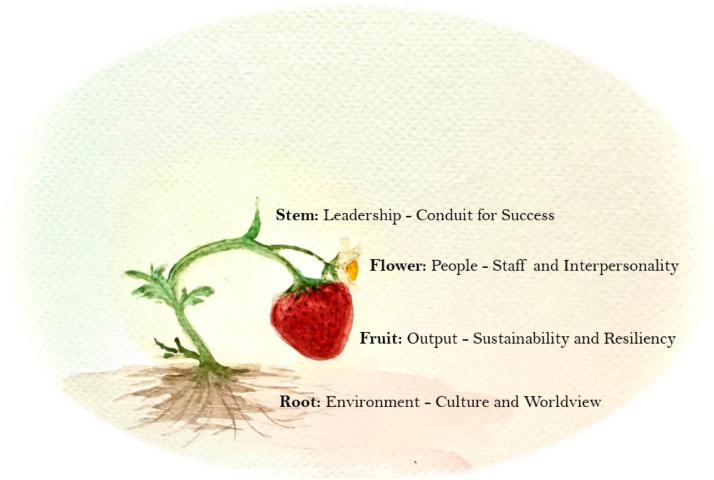
A transferable, land-based analogy to matriarchal values in leadership.

Upholding land-based analogies to promote matriarchal leadership approaches, the strawberry plant depicts the linkages of such ideas drawn in this paper. The strawberry is considered a “love plant” in some cultures, as well as a plant that represents protocols and milestones of the developing woman. Moreover, some know the strawberry as “heart medicine,” carrying teachings that the heart guides us in balancing our mind, body, spirit, and emotions. It represents caring for self and others. The strawberry is adaptable to its ecosystem, wherein its outputs, the fruit, and the quality of the berries produced by the plant are reliant on a sustainable season cycle, and adequate inputs within the soil and root system. Toxic environments, similar to those that have lateral violence or colonial disruptions will impact the output of the plant. On the other hand, plants that are fostered in a rich, generous, and well-nurtured environment, taking time to reciprocate health from its neighbouring plants and organic compounds will have a wealthy crop in the season to come. Similar to matriarchal leadership principles, the stem acts as a conduit for the nutrients of the land to make their way to the flowers that are developing above. The flowers represent the people that benefit from the nutrients fostered through the stem. The strength of the roots and the stem will determine the interpersonality of the developing plant, which can then cross-pollinate with other plants in its environment. Finally, the fruits represent the sustainable and resilient work environments that are natural outputs from strong foundations, supportive leaders, and healthy people within an organization. Plants with healthy foundations and strong stems as conduits for interactive flowers will create sweet and sustainable outputs, bearing seeds to inform sustainability of more plants Figure 1.

## Reawakening matriarchal structures in leadership

Such linkages between the past and the present as well as equity between Indigenous and westerns perspectives note the synergies that can exist in two-eyed seeing. Western dominant leadership systems have often favoured individualistic and white masculinity.^13^ However, such perspectives have demonstrated narrow-minded outcomes that can negate long-term, intersectional outcomes. Not limited to Indigenous organizations, top-down, hierarchical organizations that promote individualistic approaches to solutions tend to undermine the value of more feminist qualities in leadership such as cooperation, inclusivity, and collectivity.^13^ The absence of feminine balance in corporate and organizational models can further marginalize the marginalized, which, in British Columbia, includes those with inequitable determinants of health.

As author CMC reflects: *I see the concept of professionalism as a starting place for many to examine their values and beliefs in leadership. Professionalism is broadly viewed as behaviours that meet organizational and societal expectations. When systems are designed through settler colonial environments, those expectations may inherently discriminate against Indigenous women and 2SLGBTQ+ people. Challenging professionalism, and the behaviours and expectations that have been built into organizational structures, can help leaders to create safer spaces. I see rematriation in leadership as embracing people where they are, as they are, and nurturing their gifts. Caring for the whole person, patient, staff, and provider alike, is an act of health system transformation.*

## Conclusion

Traditional models of matriarchal leadership uphold the values of relationality between one another, our surroundings, and our larger systems. Current and ongoing acts of colonization enforce patriarchal structures at the root of our systems, many of which have been adopted by Indigenous Peoples and the organizations they operate within. As the resilience, organizational sustainability, and the natural ecosystems humans rely on for survival continue to reveal their value, feminist and Indigenous-centred leadership practices are re-awakened. Mirroring the function of the organisms that come from the land, cyclical, reciprocal, and collaborative environments for leadership can create sweet and immediate outputs as well as sustain nourishment for generations to come. The persistence of ancient systems that come from the land can continue to reinforce balance and transform the systems we surround ourselves with.
